# A lithium ion battery using an aqueous electrolyte solution

**DOI:** 10.1038/srep28421

**Published:** 2016-06-22

**Authors:** Zheng Chang, Chunyang Li, Yanfang Wang, Bingwei Chen, Lijun Fu, Yusong Zhu, Lixin Zhang, Yuping Wu, Wei Huang

**Affiliations:** 1 New Energy and Materials Laboratory (NEML), Department of Chemistry & Shanghai Key Laboratory of Molecular Catalysis and Innovative Material, Fudan University, Shanghai 200433, China; 2 College of Energy, Nanjing Tech University, Nanjing 211816, Jiangsu Province, China

## Abstract

Energy and environmental pollution have become the two major problems in today’s society. The development of green energy storage devices with good safety, high reliability, high energy density and low cost are urgently demanded. Here we report on a lithium ion battery using an aqueous electrolyte solution. It is built up by using graphite coated with gel polymer membrane and LISICON as the negative electrode, and LiFePO_4_ in aqueous solution as the positive electrode. Its average discharge voltage is up to 3.1 V and energy density based on the two electrode materials is 258 Wh kg^−1^. It will be a promising energy storage system with good safety and efficient cooling effects.

Recently, the rapid depletion of fossil fuels and the increasing emission of greenhouse gases causing air pollution have become serious issues. Development of the next generation of green energy storage devices is urgently demanded^1^,^2^,^3^. New energy industry including electric vehicles and large-scale energy storage systems (ESSs) such as smart grids requires energy storage systems with good safety, high reliability, high energy density and low cost. Hence, “three E” criteria, namely, energy (high energy densities with respect to unit weight and volume), economics (low manufacturing costs and long cycling life), and environment (safe, non-toxic and high reliability) is used to determine the suitable ESS that meets the needs of application^4^,^5^,^6^. Although lithium ion batteries (LIBs) show good promise with some quite dominant advantages over conventional batteries, their applications in large-scale ESS such as electric vehicles and smart grids are still not commercially viable due to their inherent safety problems related to the use of high cost, toxic and flammable organic electrolyte and slow charging performance^7^,^8^,^9^.

Since aqueous rechargeable lithium batteries (ARLBs) using positive electrode materials from commercial LIBs were first introduced in 1994, they have attracted wide attention as a promising system because of their low capital investment, environmental friendliness and good safety^10^,^11^,^12^. Additionally, the organic electrolyte solution is replaced by the non-flammable aqueous electrolyte with high ionic conductivity, about 2 orders of magnitude higher than those of the organic electrolytes, which can lead to good rate performance and low over potentials. Recent results show that nanostructured materials as positive electrode materials for ARLBs present much better electrochemical performance than LIBs using organic electrolytes. For example, LiMn_2_O_4_ nanotube shows a superfast second-level charge and discharge capability and excellent cycling behavior because of the nanostructure and preferred orientation^13^,^14^,^15^. Recently, we prepared a coated lithium metal, which is stable in aqueous electrolytes, to build an ARLB with a stable working voltage of 4.0 V, a break-through the window of electrochemical stability of water (1.229 V) has been achieved^16^,^17^,^18^,^19^,^20^. They can be a suitable alternative because their energy density can be above that of the corresponding lithium ion batteries. However, the use of Li metal as the negative electrode in conventional lithium metal secondary batteries are restricted by cycling due to dendrites, which can break down during the cycling and lead to sudden death of the battery. Recently, another kind of ARLB, aqueous lithium ion battery, was reported with stable voltage up to 3.0 V due to the use of ‘water-in-salt’ electrolyte^21^.

Here, we report another aqueous lithium ion battery (ALIB) which consists of graphite instead of Li metal as the negative electrode, which presents excellent cycling, and commercial LiFePO_4_ as the positive electrode in aqueous electrolyte. Its capacity is up to 121 mAh g^−1^ on the basis of LiFePO_4_ with excellent cycling performance and satisfactory rate capability.

## Results

As shown in Fig. S1 of ESI (electronic supporting information), the coated graphite acted as the negative electrode. Graphite on the copper current collector was at first coated with a gel polymer electrolyte (GPE), which was made by a composite polymer membrane PVDF (poly(vinyldifluoride)) with NWFs (nonwoven fabrics)^22^ of 40 μm saturating with the 1 mol l^−1^ LiClO_4_ solution in ethylene carbonate, diethyl carbonate and dimethyl carbonate (volumetric ratio is 1:1:1). Its ionic conductivity of Li^+^ ions is about 0.3 mS cm^−1^ at room temperature. Then, a LISICON film consisting of Li_2_O-Al_2_O_3_-SiO_2_-P_2_O_5_-TiO_2_-GeO_2_, which was bought from Ohara Inc., Japan, was further simply put on the GPE. Thickness and ionic conductivity of the LISICON film are 150 μm and 0.1 mS cm^−1^ at room temperature, respectively. If LISICON film contacts Li metal or lithiated graphite directly, some metal oxides such as GeO_2_ in the LISICON film will be reduced by Li metal leading to poor ionic conductivity. As a result, the GPE ensures the good electrochemical stability of the LISICON film. Here, the LISICON film acted as a solid separator to keep water away and allowed only the passage of Li^+^ ions. Then a passage of Li^+^ ions between the coated graphite negative electrode and the aqueous solution was built up.

**Figure 1:**
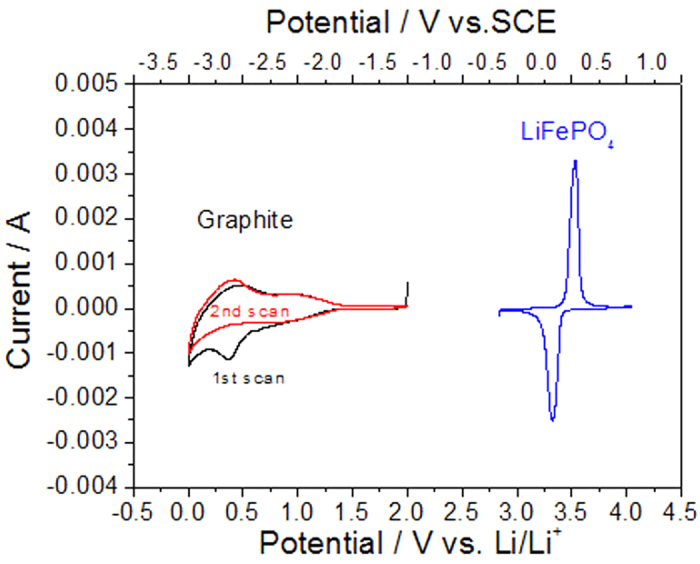
CVs for graphite negative electrode in 1 mol l^−1^ LiClO_4_ solution with ethylene carbonate, diethyl carbonate and dimethyl carbonate (volumetric ratio 1:1:1) and LiFePO_4_ positive electrode in 0.5 mol l^−1^ Li_2_SO_4_ aqueous solution by using Ni mesh and saturated calomel electrode (SCE) as the counter and the reference electrodes, respectively, at the scan rate of 0.5 mV s^−1^.

The cyclic voltammograms (CVs) of the graphite negative electrode in 1 mol l^−1^ LiClO_4_ solution and that of LiFePO_4_ in 0.5 mol l^−1^ Li_2_SO_4_ aqueous solution at the scan rate of 0.5 mV s^−1^ are shown in Fig. 1. In the case of graphite, there is a reduction peak at 0.4 V (vs. Li/Li^+^) in the first scan corresponding to the formation of SEI (solid electrolyte interface). Next, the reversible Li^+^ ion intercalation-deintercalation process occurred at about 0.2 V (vs. Li/Li^+^). In the second scan, there is only one pair of redox peaks for Li^+^ ion intercalation-deintercalation. The charge and discharge curves of graphite in the 1st, 2nd 4th and its cycling performance are shown in Fig. S2a. Similar to former reports, there is some slight irreversible capacity in the first cycle due to the formation of SEI film. However, from the second cycle, the capacity is stable and the coulomb efficiency can be about 100% with a reversible capacity of around 120 mAh g^−1^. These results are consistent with the above CV results.

Commercial LiFePO_4_ (see Fig. S3 for morphology and X-ray diffraction in ESI) is a positive electrode material for lithium-ion batteries with good thermal stability and environmental benign^23^. Its main redox peaks for the deintercalation/intercalation of Li^+^ ions in 0.5 mol l^−1^ Li_2_SO_4_ aqueous solution are located at 0.3 V and 0.1 V (vs. SCE), respectively, which is consistent with the formerly reported intercalation/deintercalation behavior of LiFePO_4_ in the aqueous electrolytes^23^. The separation of the redox peaks is much narrower than that in the organic electrolyte^24^.

Based on the above discussion, both the coated graphite and LiFePO_4_ in aqueous electrolyte provide reversible Li^+^ intercalation and deintercalation. They establish another ALIB whose electrode reactions is shown in the following equations (1–3):

Positive electrode reaction:


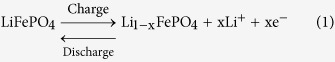


Negative electrode reaction:


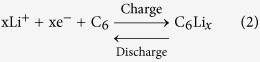


**Figure 2:**
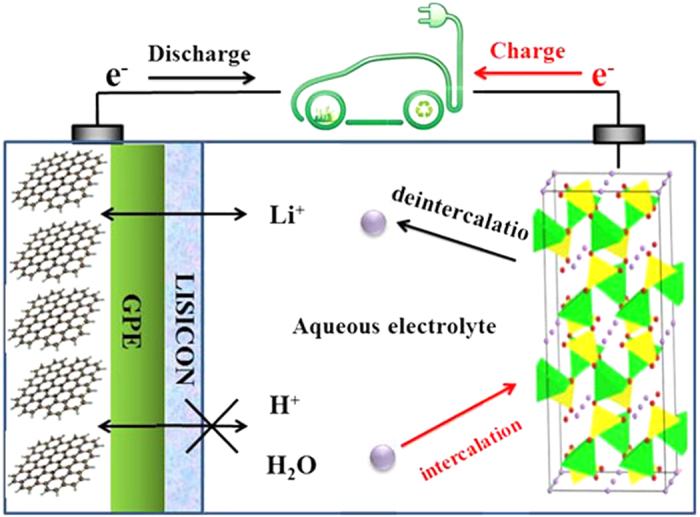
Schematic illustration of our designed ALIB using the graphite coated by GPE and LISICON as negative electrode, LiFePO_4_ in 0.5 mol l^−1^ Li_2_SO_4_ aqueous electrolyte as positive electrode.

Total reaction:


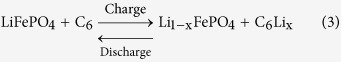


wherein x ≤ 1. During the charge process, Li^+^ ions deintercalate from the LiFePO_4_ olive structure and electrons flow via the outer circuit. The deintercalated Li^+^ ions pass through the aqueous solutions, LISICON film and GPE, and intercalate in the graphite through reduction by electrons from the outer circuit. During the discharge process, the reverse process takes place. The resulting scheme is show in Fig. 2. Since its positive electrode is put into aqueous electrolyte solution instead of an organic one, it is called an aqueous lithium ion battery (ALIB).

The CV curve of the ALIB at the scan rate of 0.1 mV s^−1^ in Fig. S4 also shows a couple of redox peaks situated at 3.43 and 3.15 V, respectively, indicating the good reversibility. Here the Li^+^ intercalation-deintercalation potential of graphite is much lower than that for hydrogen evolution. The reversible intercalation-deintercalation process will not happen in water. However, in our case, Li^+^ ions act as the charge transfer media, which can cross over the hydrogen evolution potential through LISICON and arrive at the graphite negative electrode directly. This cross-over is similar to the potential change between both sides of a cell membrane, the potential of Li^+^ ions decrease very sharply from the positive electrode to the negative one^16^. The Li^+^ ions in the positive side have higher potential and are very stable. Meanwhile, water and protons could not enter into the negative side with a low potential so that hydrogen could not be produced. As to the LiFePO_4_ positive electrode, it is stable in water since its potential is below that for the oxygen evolution and much higher than that for hydrogen evolution^18^.

**Figure 3:**
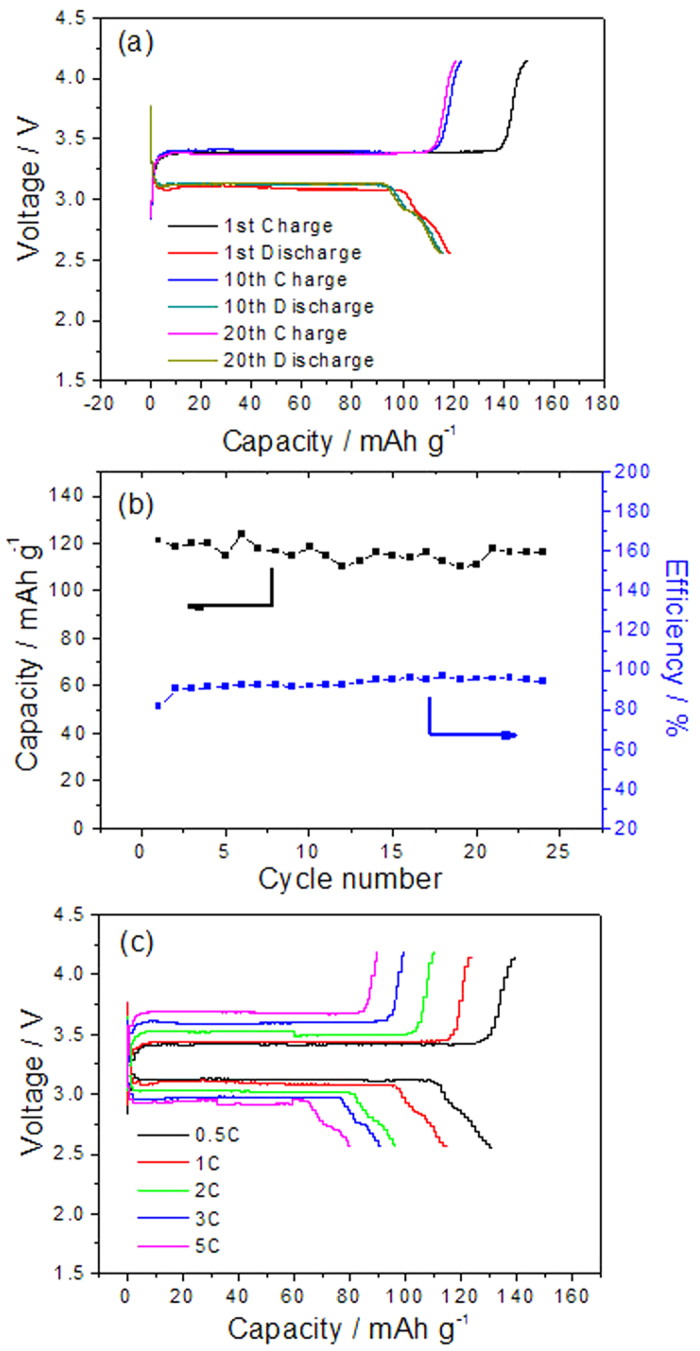
Electrochemical performance of the assembled ALIB: (**a**) Galvanostatic charge-discharge curves in the first, 10th, 20th cycles at the current density of 100 mA g^−1^ based on the positive electrode between 2.5 and 4.2 V, (**b**) the cycling performance between 2.5 and 4.1 V at the current density of 100 mA g^−1^ based on the LiFePO_4_ positive electrode, and (**c**) charge-discharge curves at different rates.

The electrochemical performance of the assembled ALIB is shown in Fig. 3. In the first cycle between 2.5 and 4.2 V at 100 mA g^−1^ based on the positive electrode (Fig. 3a), there are two distinct voltage plateaus at 3.39 and 3.11 V, respectively, which are in good agreement with the above CV results. At 100 mA g^−1^ the initial charge and discharge capacities of this battery based on the mass of LiFePO_4_ are 149 and 121 mAh g^−1^ (Fig. 3b), respectively, and the initial coulomb efficiency is 81.2%. Its discharge capacities can be comparable to those of LiFePO_4_ as positive electrode in aqueous or organic electrolytes. The charge and discharge curves in the 10th and the 20th cycles for the assembled ALIB overlap very well, indicating a good reversibility. The energy density of this ALIB is 258 W h kg^−1^ based on the cell voltage and cell capacity, which is much higher than those of previously reported ARLBs (30–45 Wh kg^−1^), whose voltages are below 2 V^14^, and that of ALIB (<120 Wh kg^−1^)^21^. If graphite with higher capacity is used, the energy density will be higher.

The assembled ALIB presents 95.8% capacity retention at the current density of 50 mA g^−1^ based on the mass of LiFePO_4_ after 24 cycles. When the rate increases, the capacity retention of this ALIB is still very good (Fig. 3c). For example, at a rate of 0.5C, 1C, 2C, 3C, 5C, the discharge capacities based on the mass of LiFePO_4_ are 131, 118, 100, 95 and 80 mAh g^−1^, respectively. Though it is poorer than that of the first generation ARLBs, it is comparable with those of LIBs.

## Discussion

Our design is different from our previous work and conventional lithium ion battery though both sides are based on the traditional intercalation/deintercalation reactions. On the one hand, we do not use the coated Li metal as the negative electrode due to the problem of its lacking stability. On the other hand, the aqueous electrolyte has higher thermal capacitance and can absorb large amounts of heat. During the same charge-discharge process, the temperature of this system will be more stable in comparison with that of the conventional lithium ion batteries. Water or aqueous electrolyte is in direct contact with both the negative and the positive electrodes, and the cooling effects are very efficient. A cooling system, which is usually required for large capacity battery modules, is not needed for the application of this battery in electric vehicles. If other intercalation compounds such as LiMn_2_O_4_, LiCoO_2_ and Li[Ni_1/3_Co_1/3_Mn_1/3_] O_2_, which are stable in aqueous electrolytes, are used as the positive electrode^25^,^26^,^27^, not only the average discharge voltage will be higher but also the energy density and cycling performance will be improved. In addition, here the composite polymer membrane is flame retarding and becomes a gel after saturating with the organic electrolyte, which presents much slower evaporation speed in comparison with the organic electrolytes^24^. This design is also different from the reported ALIB whose stable voltage is up to 3.0 V due to the large polarization or overpotentials due to the use of ‘water-in-salt’ electrolyte^21^. As a result, it will be a promising energy storage system.

However, there are some problems related to the possible practical applications by adopting the solid state electrolytes (LISICON) due to the following two reasons. (1) Its cost is high, and future methods to decrease its manufacturing cost are needed since its primary materials are not expensive; its ionic conductivity at room temperature is not high enough so that large over potential or polarization is produced. It is encouraging that many endeavours are under way^20^,^28^.

In summary, our work provides another aqueous lithium ion battery (ALIB) using graphite coated with GPE and LISICON as the negative electrode, lithium intercalation compound LiFePO_4_ in 0.5 mol l^−1^ Li_2_SO_4_ aqueous solution as the positive electrode. It is much safer than the traditional lithium ion batteries since water or aqueous electrolyte solution provides efficient cooling effects. Its average discharge voltage is 3.1 V, much higher than the window of electrochemical stability of water (1.229 V). Based on the mass of both electrodes, its energy density can be 258 Wh kg^−1^, and the cycling behaviour is satisfactory.

## Method

### Coated graphite electrode

The graphite electrode was prepared by coating the N-methyl-2- pyrrolidone (NMP)-based slurry containing the commercial graphite, acetylene black and poly- vinylidene difluoride (PVDF) in a weight ratio of 8:1:1 on copper foil (thickness: 20 μm) using a doctor-blade technique. The coated foils were dried and punched into circular pieces (d = 15 mm), which were further dried at 120 °C for 12 h under vacuum. The mass loading of graphite was about 25 mg cm^−2^. The graphite electrode was at first simply coated (like casing) by a home-made gel polymer electrolyte (GPE), whose ionic conductivity is about 0.2 mS cm^−1^ at room temperature. The GPE was made by saturating a composite polymer membrane, PVDF (poly (vinyl difluoride)) with nonwoven fabric^22^, with a thickness of 40 μm with the 1 mol l^−1^ LiClO_4_ solution in ethylene carbonate, diethyl carbonate and dimethyl carbonate (volumetric ratio is 1:1:1)^23^. Then, a LISICON film consisting of Li_2_O-Al_2_O_3_-SiO_2_-P_2_O_5_-TiO_2_-GeO_2_, which was bought from Ohara Inc., Japan, was further simply put on the GPE. Thickness and ionic conductivity of the LISICON film are 150 μm and 0.1 mS cm^−1^ at room temperature, respectively.

### Assembling of aqueous lithium ion battery (ALIB)

The commercial LiFePO_4_ was mixed with acetylene black and poly(tetrafluoroethylene) (PTFE) in a weight ratio of 8:1:1 with the help of ethanol. After drying, the mixture was pressed into a film with an active mass loading of 3.75 mg cm^−2^, then the film was cut into disks. These disks were pressed onto Ni-grid at a pressure of 10 MPa and dried at 80 °C for one night. The coated graphite and LiFePO_4_ were immersed into the 0.5 M Li_2_SO_4_ aqueous solution to make up an aqueous lithium ion battery.

### Characterization and electrochemical testing

X-ray powder diffraction (XRD) was carried out using a Bruker Analytical X-ray System with Cu Kα radiation source filtered by a thin nickel plate. Scanning electron micrographs (SEM) were obtained with a Philips XL30 scanning electron microscope. Cyclic voltammetry (CV) and galvanostatic charging/discharging were performed at room temperature on an electrochemical working station CHI600C (Chenhua, Shanghai, China) and a cell tester 2001A (Land, Wuhan, China), respectively.

## Additional Information

**How to cite this article**: Chang, Z. *et al.* A lithium ion battery using an aqueous electrolyte solution. *Sci. Rep.* **6**, 28421; doi: 10.1038/srep28421 (2016).

## Supplementary Material

Supplementary Information
