# Supplementary material for: A lithium ion battery using an aqueous electrolyte solution
Source: Sci Rep. 2016 Jun 22;6:28421. doi: 10.1038/srep28421 (PMC4916472; doi:10.1038/srep28421)
Supplement: Supplementary Information [file srep28421-s1.pdf]

A lithium ion battery using an aqueous electrolyte solution

Zheng Chang, Chunyang Li, Yanfang Wang, Bingwei Chen, Lijun Fu, Yusong Zhu*, Lixin Zhang*, Yuping Wu,* and Wei Huang

Eletronic supporting information (ESI)

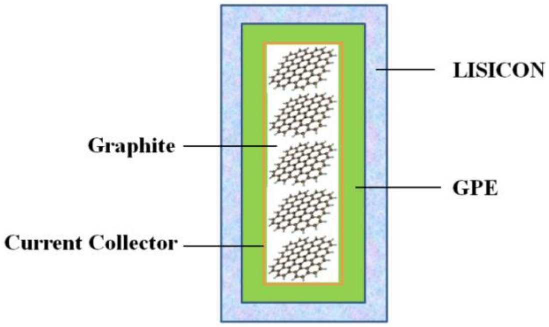

Fig. S1 The schematic structure of the coated graphite on current collector.

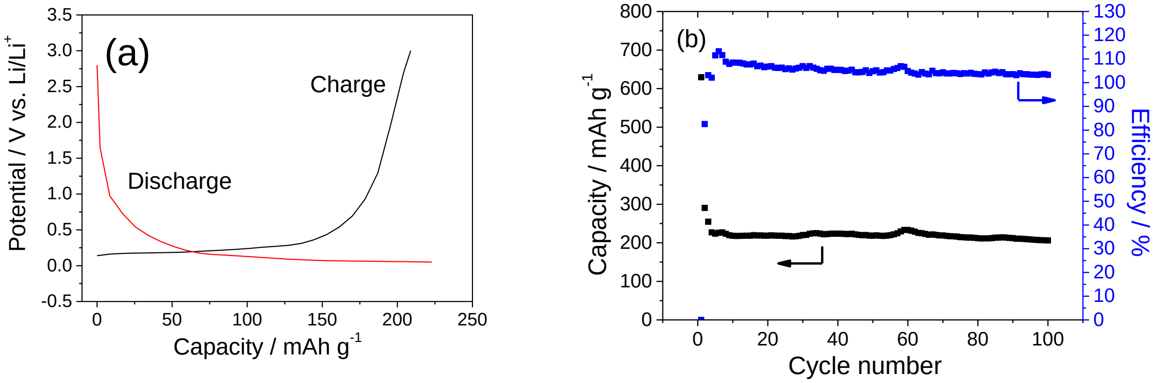

Fig. S2 (a) Typical galvanostatic charge-discharge curves and (b) the cycling performance of the graphite negative electrode at the current density of 100 mA g^{-1}.

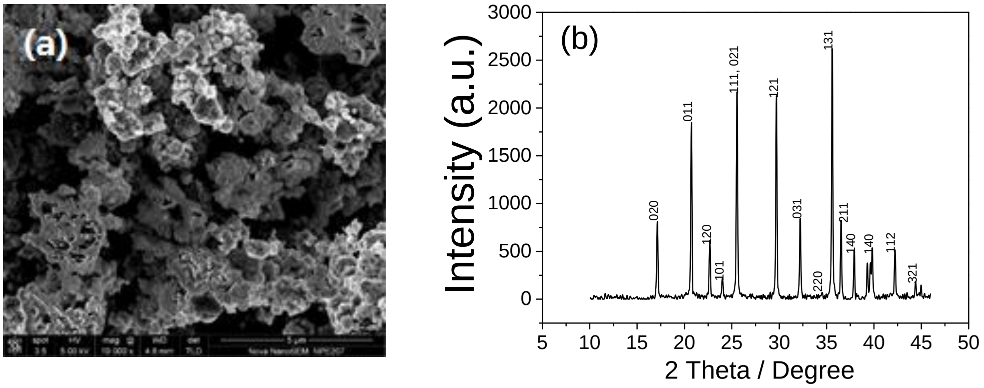

1. **Fig. S3** (a) Scan electron micrograph and (b) X-ray diffraction of $LiFePO_4$.

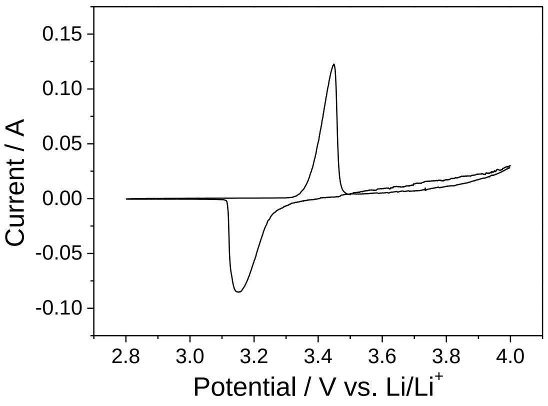

Fig. S4 Cyclic voltammogram (CV) of the assembled ALIB at the scan rate of 0.1 mV s^{-1}.
